# A Guide to the Practical Examination of the Urine

**Published:** 1891-09

**Authors:** 


A Guide to the Practical Examination of Urine. For the use of Physicians and Students. By James Tyson, M. D., Professor of Clinical Medicine in the University of Pennsylvania, and Physician to the Hospital of the University; Fellow of the College of Physicians of Philadelphia, etc., etc., etc. Seventh edition; revised and corrected, with a colored plate and wood engravings. Pp.—255. P. Blakiston, Son & Co. Philadelphia: 1891.

This little work which has just passed through six editions, has no superior in the English language. The subject matter of which it treats is complete without being verbose; it is concise, thorough, and up to date. It is a volume that should be in the hands of every medical student, and in the library of every practitioner. The excellent work usually done by the publishers is easily seen here.

